# Identifying promising GSK3β inhibitors for cancer management: a computational pipeline combining virtual screening and molecular dynamics simulations

**DOI:** 10.3389/fchem.2023.1200490

**Published:** 2023-05-22

**Authors:** Libo Hua, Farah Anjum, Alaa Shafie, Amal Adnan Ashour, Abdulraheem Ali Almalki, Ali Abdullah Alqarni, Hamsa Jameel Banjer, Sarah Abdullah Almaghrabi, Shan He, Nenggui Xu

**Affiliations:** ^1^ South China Research Center for Acupuncture and Moxibustion, Medical College of Acupuncture Moxibustion and Rehabilitation, Guangzhou University of Chinese Medicine, Guangzhou, China; ^2^ Department of Clinical Laboratory Sciences, College of Applied Medical Sciences, Taif University, Taif, Saudi Arabia; ^3^ Department of Oral and Maxillofacial Surgery and Diagnostic Sciences, Faculty of Dentistry, Taif University, Taif, Saudi Arabia; ^4^ Department of Medical Laboratory Technology, Faculty of Applied Medical Sciences, King Abdulaziz University, Jeddah, Saudi Arabia; ^5^ Center for Innovations in Personalized Medicine (CIPM), King Abdulaziz University, Jeddah, Saudi Arabia; ^6^ School of Food and Pharmacy, Zhejiang Ocean University, Zhoushan, China; ^7^ Institute for Nano Scale and Technology, College of Science and Engineering, Flinders University, Bedford Park, SA, Australia; ^8^ College of Engineering, Information Technology and Environment, Charles Darwin University, Darwin, NT, Australia

**Keywords:** glycogen synthase kinase-3, cancer, virtual screening, molecular dynamics, drug-likeness

## Abstract

Glycogen synthase kinase-3 (GSK3β), a serine/threonine protein kinase, has been discovered as a novel target for anticancer drugs. Although GSK3β is involved in multiple pathways linked to the etiology of various cancers, no specific GSK3β inhibitor has been authorized for cancer therapy. Most of its inhibitors have toxicity effects therefore, there is a need to develop safe and more potent inhibitors. In this study, a library of 4,222 anti-cancer compounds underwent rigorous computational screening to identify potential candidates for targeting the binding pocket of GSK3β. The screening process involved various stages, including docking-based virtual screening, physicochemical and ADMET analysis, and molecular dynamics simulations. Ultimately, two hit compounds, BMS-754807 and GSK429286A, were identified as having high binding affinities to GSK3β. BMS-754807 and GSK429286A exhibited binding affinities of −11.9, and −9.8 kcal/mol, respectively, which were greater than that of the positive control (−7.6 kcal/mol). Further, molecular dynamics simulations for 100 ns were employed to optimize the interaction between the compounds and GSK3β, and the simulations demonstrated that the interaction was stable and consistent throughout the study. These hits were also anticipated to have good drug-like properties. Finally, this study suggests that BMS-754807 and GSK429286A may undergo experimental validation to evaluate their potential as cancer treatments in clinical settings.

## 1 Introduction

Cancer, a hyperproliferative condition, is characterized by excessive cell division and, eventually, metastasis. Protein kinases are essential regulators of many biological processes and are targets for a wide range of human disorders (Greten and Grivennikov, 2019). Glycogen synthase kinase-3 (GSK3β), a serine/threonine protein kinase, has been discovered as a novel target for anticancer drugs. It was originally thought to be the most significant enzyme involved in the metabolism of glycogen, but it is now largely recognized as a regulator of various cellular processes, such as the activity of several metabolic and signaling proteins when it phosphorylates (Cohen and Frame, 2001; Dickey et al., 2011). It promotes tumor cell survival in several cancers by using different pro-survival pathways regulated by NF-κB (Zhang et al., 2014; Saud et al., 2016), Hh/Gli (Trnski et al., 2015), mTOR (Pal et al., 2014), and STAT3 (Gao et al., 2017). GSK3β inhibitors are now being used to treat a variety of conditions, including Alzheimer’s disease, diabetes, and cancer (Klamer et al., 2010; Medina and Avila, 2010; Zeng et al., 2014). Several inhibitors of GSK3β have been developed and progressed to early-stage clinical trials for various types of cancer (Sahin et al., 2019). One such inhibitor is Tideglusib, which was initially developed to target tau phosphorylation in Alzheimer’s disease. However, studies have shown that it can increase proapoptotic proteins in murine models of human neuroblastoma, indicating its potential efficacy in cancer treatment (Mathuram et al., 2016). Another GSK3β inhibitor, LY2090314, has demonstrated antiproliferative properties in preclinical studies involving melanoma and neuroblastoma. This ATP-competitive inhibitor has shown promising results in clinical trials for cancer treatment (Palomo and Martinez, 2017; Kunnimalaiyaan et al., 2018). Additionally, Solasodine, a naturally occurring aglycone of glycoalkaloid, has been shown to inhibit the GSK-3 pathway and induce apoptosis in various types of malignancies (Zhuang et al., 2017). Although GSK3β is involved in multiple pathways linked to the etiology of various cancers, no specific GSK3β inhibitor has been authorized for cancer therapy.

GSK3β is a 433-residue protein with three different structural domains. The first 134 residues form a 7-strand beta-barrel in the N-terminal domain. Residues 135–151 form a brief linker connecting the N-terminal domain to the alpha-helical domain. Residues 152–342 comprise the alpha-helical domain, and residues 343–433 make up the C-terminal domain. The ATP-binding site is located between the N-terminal and alpha-helical domains (Jacobs et al., 2012).

Drug development is a multidisciplinary, costly, and time-taking process. Computer-assisted drug discovery (CADD) is a constructive approach to drug development that hastens the process and decreases expenses. By reducing the need for animal models in pharmacological research and aiding in the rational design of safe drug candidates, CADD supports medicinal chemists and pharmacologists throughout drug discovery (Paul et al., 2021). The use of CADD has proven crucial to several projects across a range of contexts and research environments. CADD has played a substantial role in the identification and optimization of successful compounds that have moved to further stages of the drug development pipeline or commercialization (Talele et al., 2010). CADD has limitations due to the accuracy of computational models, the scarcity of structural data, the limited chemical diversity, the complexity of drug targets, and the lack of experimental validation (Sliwoski et al., 2014).

To identify possible candidates for targeting the binding pocket of GSK3β, we employed computational approaches to screening a diverse library of therapeutically active potential candidates for targeting the binding pocket of GSK3β.

## 2 Methodology

### 2.1 Retrieval and preparation of GSK3β protein

The GSK3β protein (PDB: 4AFJ) was retrieved from the PDB database (Gentile et al., 2012). Heteroatoms, water molecules, and co-crystallized ligands were extracted, and the protein was saved in.pdb format. The heteroatoms were proto-oncogene frat, a 30 amino acid short peptide, SO4, GOL, and SJJ. The clean protein was finally prepared using Discovery Studio 2021 for further studies.

### 2.2 Compound library preparation and virtual screening

The process of identifying new compounds with specific bioactivity has been transformed by virtual screening methods, which use computer simulations to assess large structure libraries against a biological target (Macalino et al., 2015). This study employed a library of 4,222 anti-cancer compounds (retrieved from https://www.selleckchem.com), including both FDA-approved drugs and naturally occurring substances. The collection included compounds that were cell-permeable, therapeutically active, and diverse in terms of their chemical structure. The compound library was downloaded in ‘sdf’ format and then processed in ‘PyRx 0.8’ program (Dallakyan and Olson, 2015). The compounds library was minimized utilizing ‘UFF’ force field as the energy minimization parameter and finally saved in the ‘pdbqt’ format for further analysis. The grid coordinates of the GSK3β were set as X = 104.048, Y = 26.822, and Z = −12.474. The best hits were carefully chosen based on the binding affinity and interaction analysis toward GSK3β.

### 2.3 Physicochemical and ADMET properties

The DataWarrior tool was used to predict the drug-likeness and physicochemical properties of the top ten compounds, and the ProTox-II (Banerjee et al., 2018) and pkCSM web servers (Pires et al., 2015) were used for ADMET and pharmacokinetic properties.

### 2.4 MD simulations

The GROMACS 2021.4 software and GROMOS96 43a1 force-field were used to perform MD simulations of three complexes: GSK3β-control, GSK3β-BMS-754807, and GSK3β-GSK429286A, all at 300 K (Pol-Fachin et al., 2009). The topology and force-field factors of the compounds were generated using the PRODRG server, and their atoms were combined in the complex topology files. Na^+^ and Cl^−^ ions were introduced to neutralize the charges on the GSK3β protein complexes using the ‘gmx_genion’ module (0.15 M) (Schuttelkopf and van Aalten, 2004). The ‘particle-mesh Ewald’ method was employed to analyze the interactions of GSK3β with these selected compounds. The system (for MD simulation) was minimized employing the ‘steepest descent’ method (1,500 steps) and equilibrated over a 100-ps period at constant volume in two stages: NVT and NPT ensembles. The total simulation of 100 ns was conducted at 300 K. Trajectories were analyzed using GROMACS modules, and 3D models were created using VMD (Humphrey et al., 1996) and PyMOL (Yuan et al., 2017).

## 3 Results and discussion

GSK3β dysfunction has been reported in various cancer types (Domoto et al., 2020), and has been identified as being at the crossroads of various biochemical pathways, including cancer-related pathways (Duda et al., 2020). Here in this study, a unique collection of 4,222 anti-cancer compounds were screened against the active pocket of GSK3β. Based on binding affinity, we chose the top ten screened compounds for further visual inspection and interaction studies (Table 1).

**TABLE 1:** Binding affinity of top 10 screened compounds.

S. No	Ligand	Binding affinity (kcal/mol)
1	YM201636	−10.6
2	OSI-906	−10.3
3	BMS-754807	−10.2
4	Tipifarnib	−10
5	VX-809	−10
6	INCB28060	−9.8
7	GSK429286A	−9.5
8	Limonin	−9.1
9	Icotinib	−8.7
10	AZ628	−8
11	AR-AO-14418 (Positive control)	−7.5

The drug-likeness and physicochemical attributes of these top 10 compounds were anticipated using the DataWarrior tool, which employs several parameters including LogP, LogS, H-bond donors and acceptors, relative PSA, and the presence of structures with particular pharmacological properties. Both GSK429286A and BMS-754807 retain an adequate range of drug-likeness properties (Table 2). GSK429286A was discovered to be a selective inhibitor of Rho-associated coiled-coil protein kinase 1 (ROCK1) and ROCK2. These kinases are involved in a several cellular activities, which include cell motility, contraction, and adhesion, and have been associated to cancer, cardiovascular disease, and neurological disorders (Kim et al., 2021). BMS-754807 efficiently and irreversibly inhibits both insulin receptor (IR) family kinases and the insulin-like growth factor 1 receptor (IGF-1R). These kinases have a high binding affinity for it (Ki, 2 nmol/L), which is important for controlling cell growth, survival, and metabolism. BMS-754807 is now in phase I clinical trials for the treatment of multiple types of human cancer (Carboni et al., 2009).

**TABLE 2:** Physicochemical assessment of top 10 screened compounds.

Molecule name	Mol Weight	cLogP	cLogS	H-acceptors	H-donors	Relative PSA	Drug likeness	Mut	Tum	Rep	Irr
Icotinib	391.41	2.9067	−3.673	7	1	0.23718	−12.415	NO	NO	NO	NO
INCB28060	412.41	2.2964	−3.284	7	1	0.24682	4.3136	NO	NO	NO	NO
OSI-906	421.49	4.0356	−6.927	6	2	0.2212	1.7312	NO	NO	NO	NO
GSK429286A	432.37	3.3102	−5.084	6	3	0.24809	−1.5348	NO	NO	NO	NO
AZ628	451.51	4.6632	−6.584	7	2	0.21932	−3.6106	NO	NO	NO	NO
VX-809	452.40	5.632	−6.513	7	2	0.26229	−1.4961	NO	NO	NO	NO
BMS-754807	461.49	2.3302	−5.859	10	3	0.31262	6.8809	NO	NO	NO	NO
YM201636	467.47	2.7535	−7.308	10	2	0.32145	2.2644	NO	NO	NO	NO
Limonin	470.51	1.0279	−4.073	8	0	0.32001	−3.0035	NO	NO	NO	NO
Tipifarnib	489.39	4.03	−5.185	5	1	0.14038	2.4006	NO	NO	NO	NO

**FIGURE 1:**
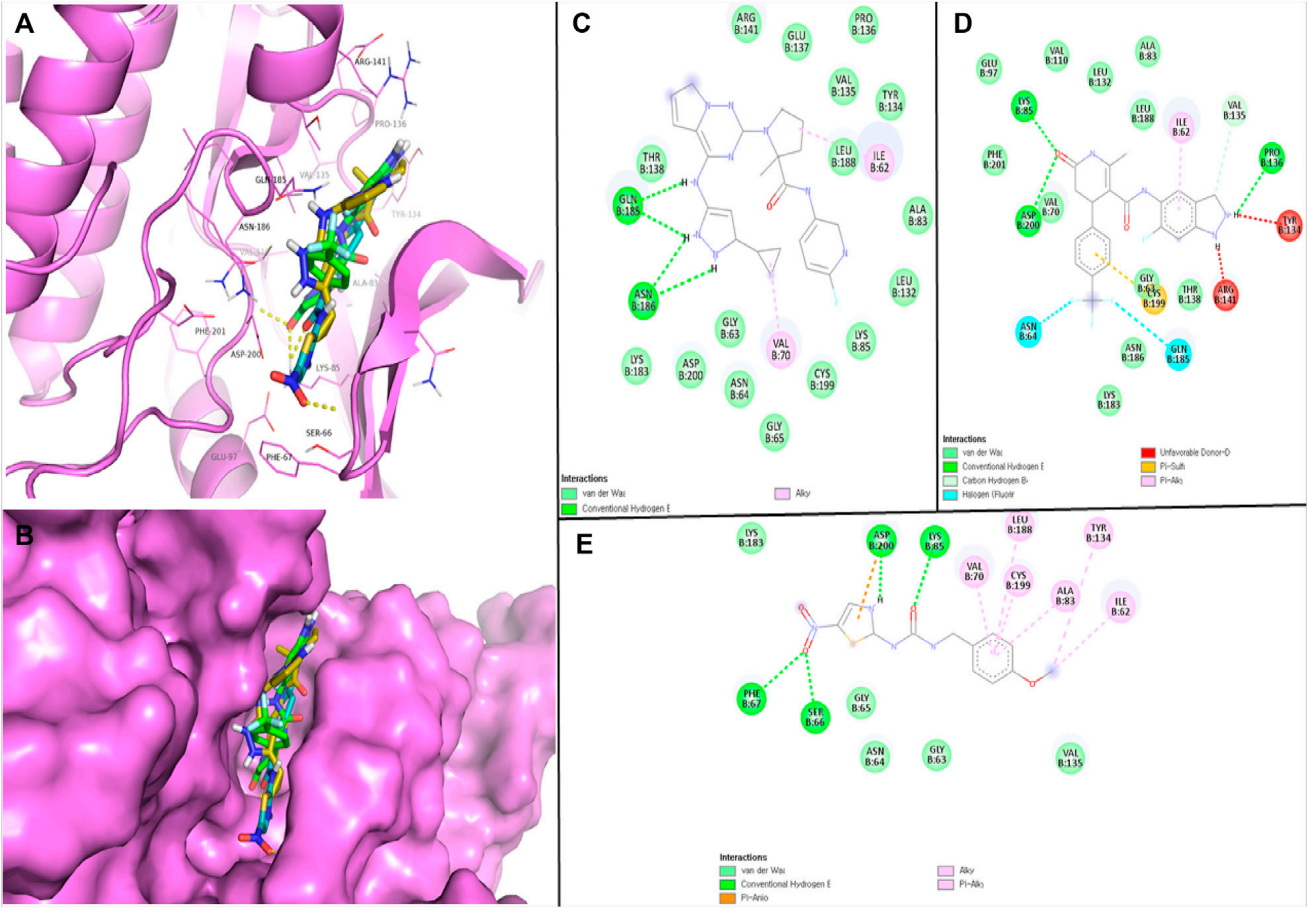
Visualization of hits and positive control in the GSK3β binding pocket **(A, B)**; 2D view of GSK3β residues interacting with BMS-754807 **(C)**, GSK429286A **(D)**, and AR-AO-14418 **(E)**.

The robust stability of the hit compounds within the GSK3β active site was attributed to the presence of various Van der Waals and H-bonding interactions (Table 3). These interactions aided in the intercalation of the compounds into the active site, increasing their binding affinity and overall stability (Figures 1A–E). BMS-754807 interacted with several residues like Ile62, Gly63, Asn64, Gly65, Val70, Ala83, Lys85, Leu132, Tyr134, Val135, Pro136, Glu137, Thr138, Arg141, Lys183, Gln185, Asn186, Leu188, Cys199, and Asp200 and residues of GSK3β. The Van der Waals interaction involves a set of amino acid residues, namely, Gly63, Asn64, Gly65, Ala83, Lys85, Leu132, Tyr134, Val135, Pro136, Glu137, Thr138, Arg141, Lys183, Leu188, Cys199, and Asp200; while Gln185 and Asn186 residues were involved in H-bonding (Figure 1C). Further, GSK429286A interacted with Ile62, Gly63, Asn64, Ala83, Lys85, Glu97, Val110, Leu132, Tyr134, Val135, Pro136, Thr138, Arg141, Val70, Lys183, Gln185, Asn186, Leu188, Cys199, Asp200, and Phe201 residues of GSK3β. Gly63, Val70, Ala83, Lys85, Glu97, Val110, Leu132, Val135, Thr138, Lys183, Asn186, Leu188, and Phe201 residues participates in Van der Waals interactions; while Lys85, Pro136, and Asp200 residues were involved in H-bonding (Figure 1D). Several amino acid residues of the GSK3β have alrady been described to play an essential role in inhibitor binding. These residues include Ile62, Gly63, Asn64, Val70, Ala83, Lys85, Glu97, Leu132, Tyr134, Val135, Leu188, Cys199, Asp200, and Phe201 (Mishra et al., 2019). It is noteworthy that the compounds (BMS-754807, and GSK429286A) have been observed to bind with these GSK3β residues.

**TABLE 3:** H-bonded and other interactions residues.

Compounds	H-bonded residues	Number of H-bond	Other interactions
BMS-754807	Asn186, and Gln185	2	Ile62, Gly63, Asn64, Gly65, Val70, Ala83, Lys85, Leu132, Tyr134, Val135, Pro136, Glu137, Thr138, Arg141, Lys183, Leu188, Cys199, and Asp200
GSK429286A	Lys85, Pro136, and Asp200	3	Ile62, Gly63, Asn64, Ala83, Glu97, Val110, Leu132, Tyr134, Val135, Thr138, Arg141, Val70, Lys183, Gln185, Asn186, Leu188, Cys199, and Phe201
AR-AO-14418	Ser66, Phe67, Lys85, and Asp200	4	Ile62, Gly63, Asn64, Gly65, Val70, Ala83, Tyr134, Val135, Lys183, Leu188, and Cys199

N-(4-methoxybenzyl)-N0-(5-nitro-1,3-thiazol-2-yl) urea (AR-AO-14418) is a selective inhibitor of GSK3β (Bhat et al., 2003), and was used as a positive control in this study. *In vitro*, ARA014418 inhibited GSK3β without significantly inhibiting other kinases, reducing tau phosphorylation at Ser-396. *In vivo*, it induced antidepressant-like effects in rats by decreasing immobility time and both spontaneous and amphetamine-induced activity (Gould et al., 2004). AR-AO-14418 interacted with Ile62, Gly63, Asn64, Gly65, Ser66, Phe67, Val70, Ala83, Lys85, Tyr134, Val135, Lys183, Leu188, Cys199, and Asp200 residues of GSK3β (Figure 1E). Interestingly, Ile62, Gly63, Asn64, Val70, Ala83, Lys85, Tyr134, Val135, Lys183, Leu188, Cys199, and Asp200 GSK3β residues were observed to be common interacting residues with both the hits (BMS-754807, and GSK429286A) as well as the AR-AO-14418 (Figure 1C-1E). In addition, Lys85, and Asp200 were the common H-bonded residues with GSK429286A and the AR-AO-14418.

**FIGURE 2:**
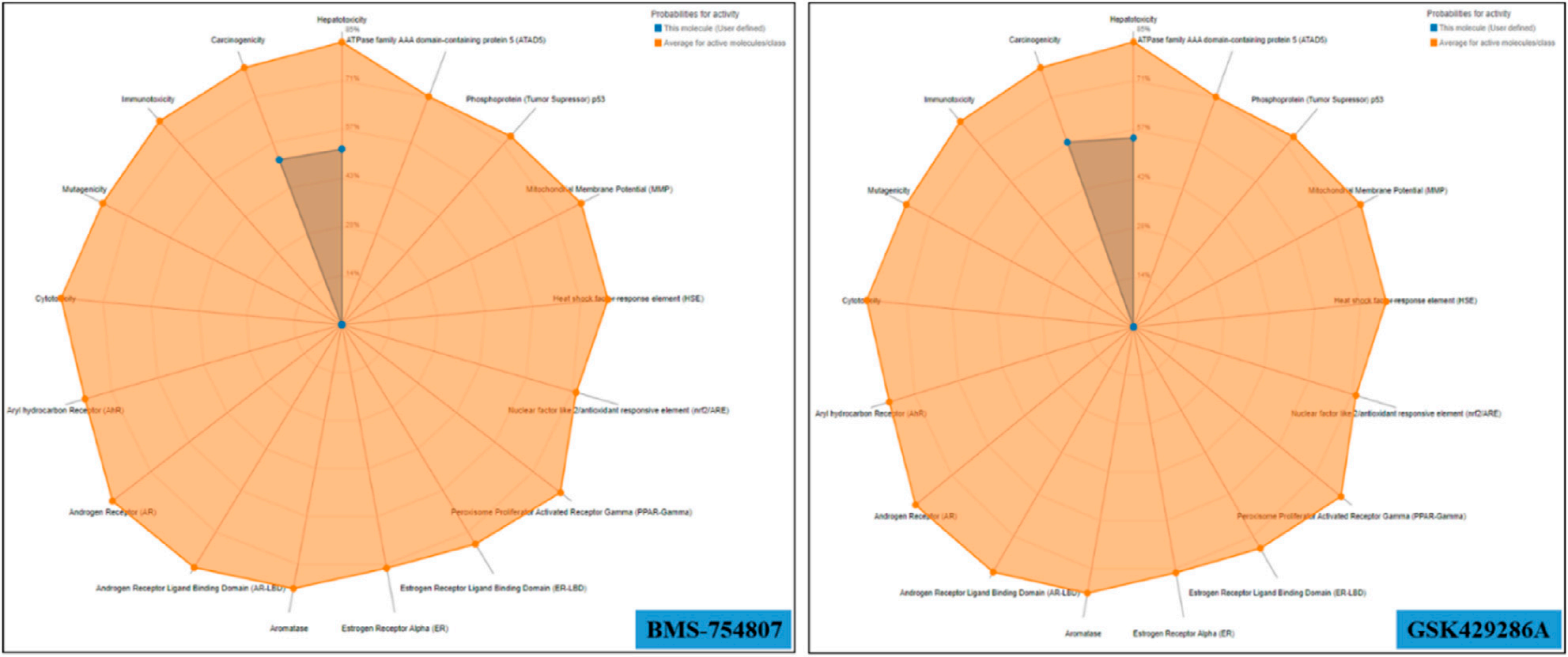
Toxicity radar chart of BMS-754807 and GSK429286A.

BMS-754807 oral toxicity prediction results were as follows: pLD50: 500 mg/kg, pToxicity Class: 4, average similarity: 39.97%, pAccuracy: 23%; while GSK429286A oral toxicity prediction results were as follows: pLD50: 50 mg/kg, pToxicity Class: 2, Average similarity: 45.44%, pAccuracy: 54.26%. BMS-754807 falls into the category of ‘harmful if swallowed’ while GSK429286A falls into the category of ‘fatal if swallowed’ as its LD50 value is greater than that of BMS-754807. In addition, multiple toxicity endpoints, including acute toxicity, hepatotoxicity, cytotoxicity, carcinogenicity, mutagenicity, immunotoxicity, *etc.*, were within the acceptable range for these two compounds (Figure 2). Further, multiple pharmacokinetic properties including ADMET of both GSK429286A and BMS-754807 were predicted by the pkCSM web server, which results that both compounds are satisfactorily appropriate in several parameters of ADMET (Table 4).

**TABLE 4:** ADMET calculation of GSK429286 A and BMS-754807.

Property	Model name			Predicted value		Unit
				GSK429286A	BMS-754807	
Absorption	Water sol			−4.068	−2.92	log mol/L
	Caco2 per			0.699	1.256	log Papp in 10–6 cm/s
	Intestinal abs			88.306	87.281	% Absorbed
	Skin Per			−2.761	−2.735	log Kp
	P-glycoprotein (P-gp) substrate			Y	Y	
	P-gp I inhibitor			Y	N	
	P-gp II inhibitor			Y	N	
Distribution	VDss (human)			−0.148	0.847	log L/kg
	Fraction unbound			0	0.143	Fu
	Per		−1.129	−1.747	−0.528	log BB
			−2.193	−3.615	−1.665	log PS
Metabolism	substrate	N		N	N	
		Y		N	Y	
	inhibitor	N		N	N	
		Y		N	N	
		Y		N	N	
		N		N	N	
		Y		N	N	
Excretion	Total Clearance			−0.02	−0.393	log mL/min/kg
	Renal OCT2 substrate			N	N	
Toxicity	AMES toxicity			N	N	
	Max. Tolerated dose (human)			−0.205	0.738	log mg/kg/day
	inhibitor	N		N	N	
		Y		Y	N	
	LD50			2.192	2.446	mol/kg
	LOAEL			1.717	1.859	log mg/kg_bw/day
	Hepato			Y	Y	
	Skin Sensitization			N	N	
	T. Pyriformis			0.349	0.285	log mM
	MinNw			1.501	1.63	

(per. = permeability; sol. = solubility; Y=Yes; N=No).

**FIGURE 3:**
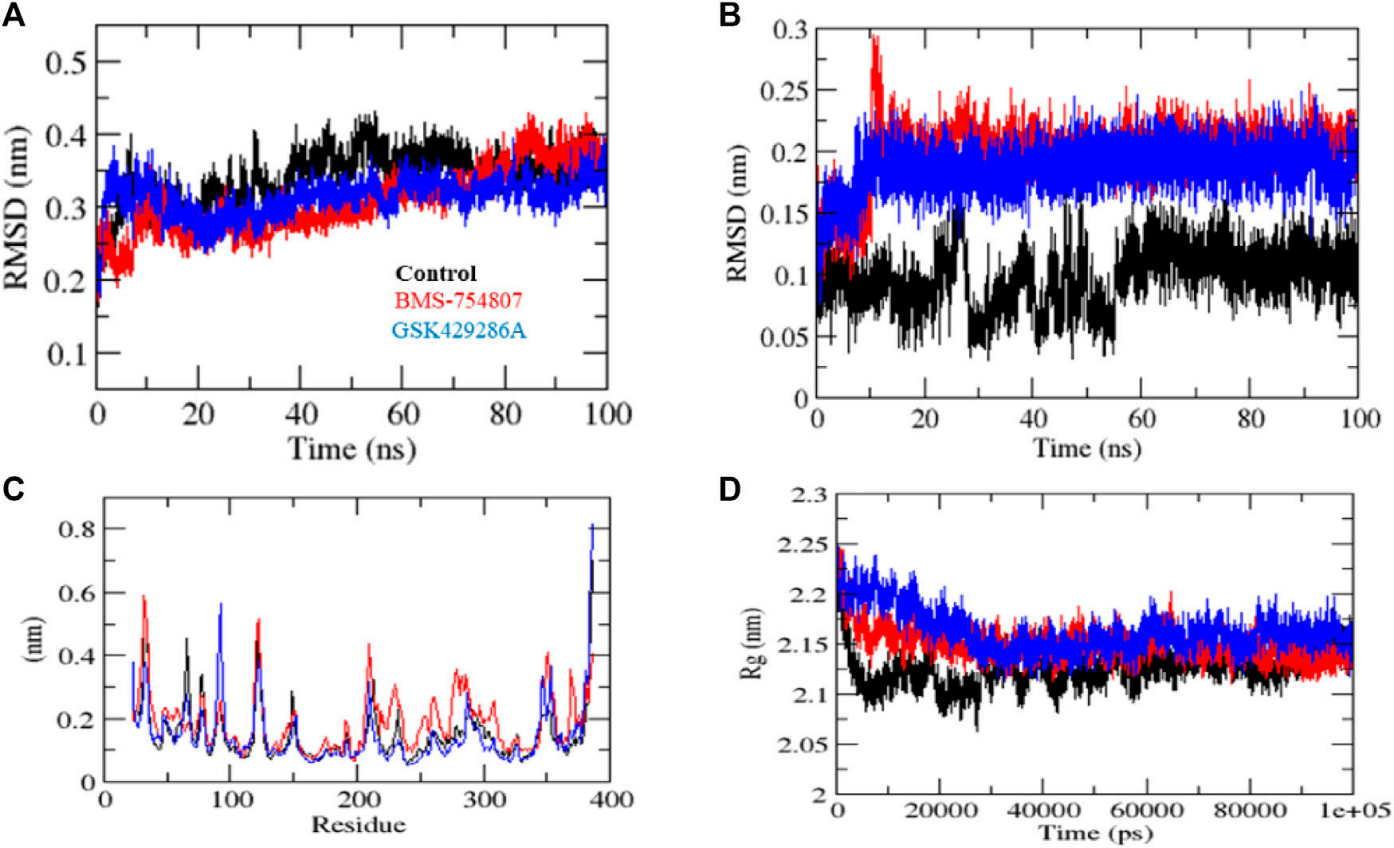
MD simulation studies of complexes. RMSD plot **(A)**, RMSD plot of ligands **(B)**, RMSF plot **(C)**, and Rg plot **(D)** of GSK3β protein with the ligands.

MD simulation studies were carried out to evaluate complex stability. Protein stability can be measured using the root mean square deviation (RMSD), where a lower RMSD value indicates a more stable protein structure. GSK3β-control, GSK3β-BMS-754807, and GSK3β-GSK429286A had RMSD average values of 0.42, 0.31, and 0.35 nm, respectively. The RMSD plot revealed that GSK3β-BMS-754807 and GSK3β-GSK429286A complex showed more binding stability than the control compound. The ‘GSK3β-control’ complex showed high deviation from its original conformation, it showed that the active site pocket of GSK3β formed quite stable interaction with both of the selected compounds. In addition, the ligand RMSD exhibits GSK3β-BMS-754807 and GSK3β-GSK429286A high deviation, and interestingly, the GSK3β-control complex showed low deviation (Figure 3A-B).

The fluctuation of each residue during the simulation was averaged, and the root mean square fluctuation (RMSF) of GSK3β was calculated while binding to GSK3β-control, GSK3β-BMS-754807, and GSK3β-GSK429286A. These values were plotted against the residue numbers of GSK3β. The GSK3β-control and GSK3β-GSK429286A backbones presented steady fluctuations in the catalytic pocket of GSK3β, presumably due to different orientations and the GSK3β-BMS-754807 complex indicated high fluctuation in region 230–260 residues (Figure 3C). On the other hand, GSK3β-control, and GSK3β-GSK429286A complexes showed the least overall fluctuations.

By measuring the distribution of atoms around the axis of a protein, the radius of gyration (Rg) provides insight into the compactness profile of a complex in a biological system. The GSK3β-control, GSK3β-BMS-754807 and GSK3β-GSK429286A complexes had average Rg values of 2.13, 2.15, and 2.17 nm, respectively. Rg plot showed lesser compactness in GSK3β-control and GSK3β-BMS-754807 than GSK3β-GSK429286A complexes. It contingent that after binding, these compounds make GSK3β stable, due to GSK3β showing less Rg trajectories (Figure 3D). Among both compounds, BMS-754807 showed better stability in the catalytic pocket of GSK3β.

**FIGURE 4:**
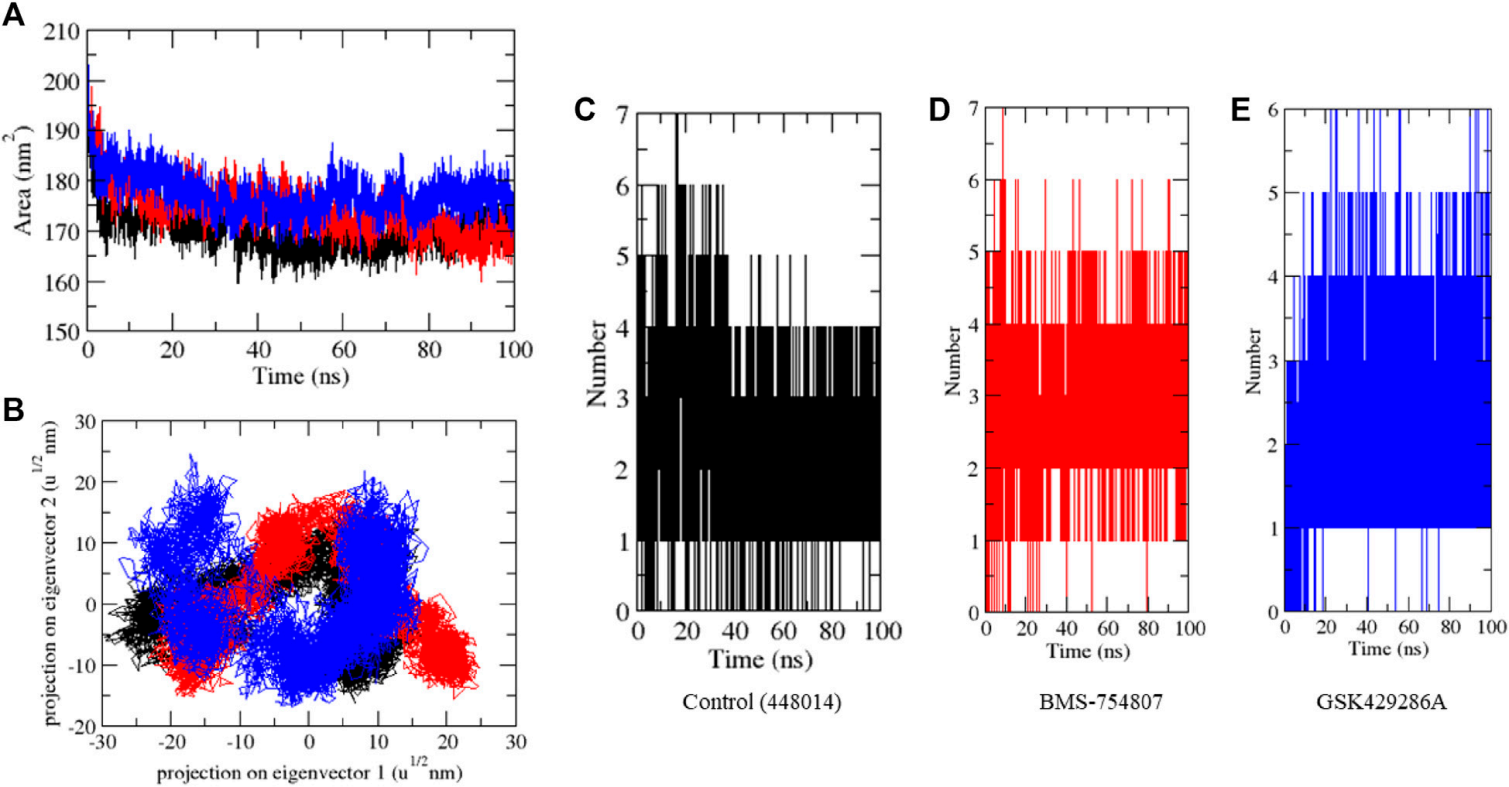
SASA plot **(A)**, 2D projection of complexes **(B)**, and number of H-bonds in complexes **(C–E)**.

The Solvent-accessible surface area (SASA) of a protein refers to the portion of its surface area that interacts with its surrounding solvent molecules. The average SASA values for GSK3β-control, GSK3β-BMS-754807, and GSK3β-GSK429286A complexes were plotted during the 100 ns simulation. The SASA values for the GSK3β-control, GSK3β-BMS-754807, and GSK3β-GSK429286A complexes were 170.51, 178.10, and 180.42 nm^2^, respectively (Figure 4A). Further, GSK3β-control and GSK3β-BMS-754807 overlapped each other in 2D projection analysis, whereas GSK3β-GSK429286A showed a different pattern (Figure 4B). SASA exploration indicated that upon binding of control, BMS-754807, surface exposure has been reduced and the GSK429286A compound increases the surface area of solvent accessibility. Further, hydrogen bond analysis was performed of the docked complexes. To evaluate the stability of the docked complexes, 100 ns simulations of GSK3β-control, GSK3β-BMS-754807, and GSK3β-GSK429286A were conducted in the presence of a solvent environment. The control and BMS-754807 compound showed an average 2–7 H-bond with GSK3β protein whereas, the GSK429286A compound showed 2–6 H-bond. It inferred that the BMS-754807 compound showed more stable interaction and might work as a potential drug against the GSK3β protein (Figure 4C-E).

**FIGURE 5:**
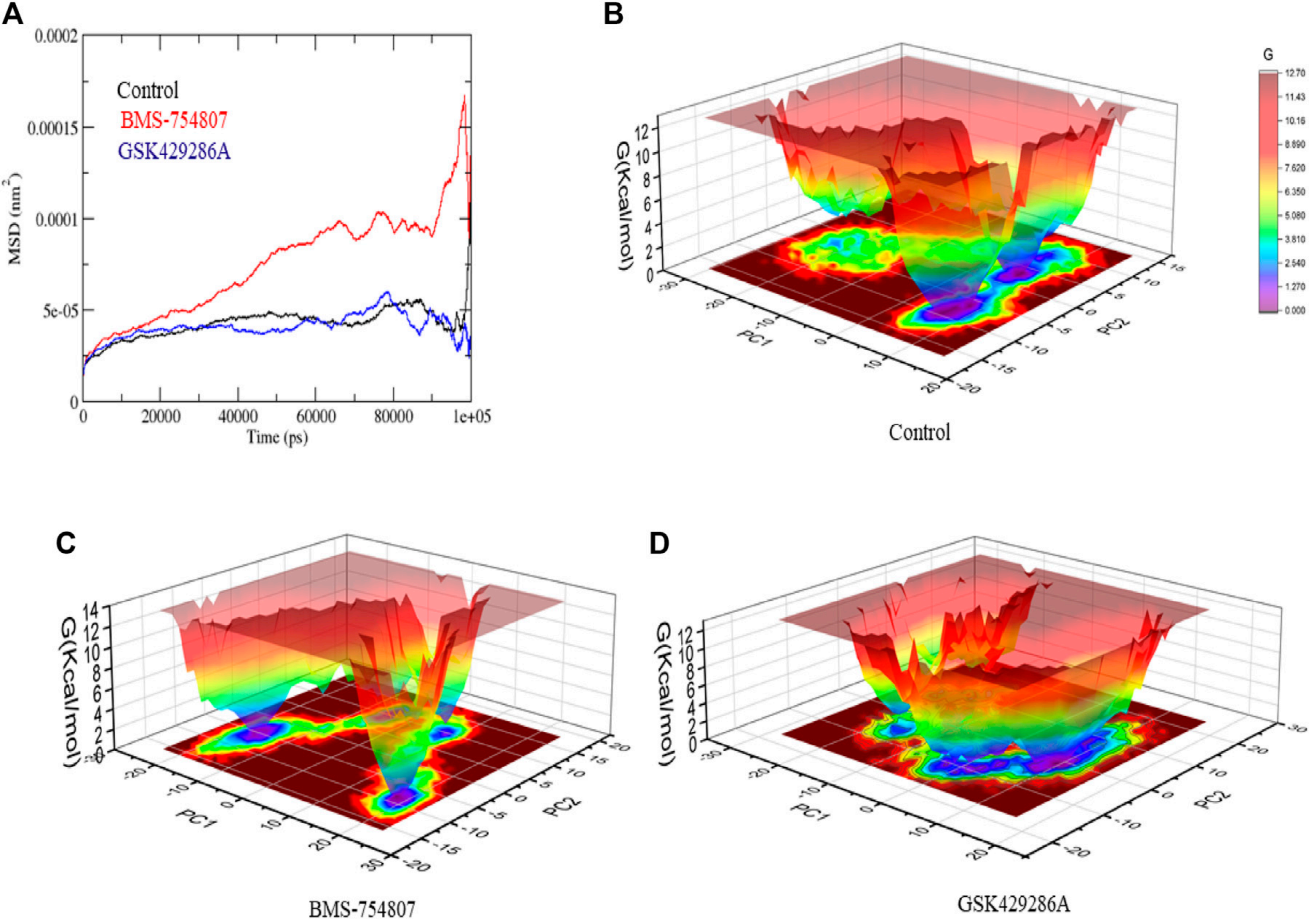
Mean square displacement plot of complexes **(A)**, GFE landscape plot **(B–D)**.

Next, Mean square displacement (MSD) was determined. It found that the GSK3β-BMS-754807 complex had a higher displacement than the control and the GSK3β-GSK429286A complex (Figure 5A). The Gibbs’ free energy (GFE) landscape was computed using GROMACS analysis modules, and the first (PC1) and second (PC2) eigenvectors were projected to generate a Comparable GFE contour map, where darker blue shades indicate lower energy levels. During the simulations, the global minima of GSK3β fluctuated due to the binding of the complexes to the GSK3β protein. The GSK3β-control and GSK3β-GSK429286A were showing similar projections; and GSK3β-BMS-754807 was showing dissimilar global minima, demonstrating that BMS-754807 global minima drastically changed during the simulation (Figure 5B-D). The above results suggested that the BMS-754807 compound might be used as a possible drug for the GSK3β protein.

Several small-molecule inhibitors of GSK3β, such as CHIR-99021, CHIR-98014, SB216763, SB415286, AR-A011418, CG701338, and CG202796, have been utilized in preclinical studies involving cell and animal models to investigate the potential involvement of GSK3β in cancer pathogenesis (Walz et al., 2017). These compounds, however, are primarily classified as “toolkit compounds” due to a lack of adequate ADMET properties required for advancement as drug candidates to clinical trials. To date, clinical trials on GSK3β inhibitors, including tideglusib and LY2090314, have demonstrated their tolerability, indicating that concerns about GSK3β inhibition causing widespread metabolic toxicity were not justified. LY2090314 has a suboptimal pharmacokinetic profile, so the lack of toxicity observed could be attributed to inadequate systemic exposure (Zamek-Gliszczynski et al., 2013). The selected compounds in this study demonstrated promising drug-like properties and have been proposed to inhibit cancer progression via their interaction with GSK3β.

## 4 Conclusion

In this study, an insilico screening approach was employed to investigate potential anti-cancer compounds targeting the GSK3β protein. BMS-754807 and GSK429286A were discovered to have high binding affinity and stability to the GSK3β protein. The favorable interactions were found to be attributed to various Van der Waals forces and H-bonding interactions. Additionally, both compounds exhibited promising drug-like properties. These findings provide a basis for further investigation *in vitro* and *in vivo* to develop potent GSK3β inhibitors for cancer management.

## Data Availability

The original contributions presented in the study are included in the article, further inquiries can be directed to the corresponding authors.
